# Serum steroid hormone profiles in reproductive-age women with systemic lupus erythematosus: associations with clinical manifestations and disease activity

**DOI:** 10.3389/fimmu.2026.1755060

**Published:** 2026-04-22

**Authors:** Xiaotong Chen, Gaowen Zhang, Juan Cai, Haining Song, Bohan Chang, Huiyan Li, Pingting Yang

**Affiliations:** 1 Department of Rheumatology and Immunology, the First Hospital of China Medical University, Shenyang, China; 2 Department of Thoracic Surgery, Qingdao Hospital, University of Health and Rehabilitation Sciences(Qingdao Municipal Hospital), Qingdao, China; 3 Department of Rheumatology and Immunology, Chifeng Municipal Hospital, Chifeng, China

**Keywords:** aldosterone, cortisol, hypothalamic-pituitary-adrenal axis, steroid hormones, systemic lupus erythematosus

## Abstract

**Objective:**

To characterize the serum steroid hormone profile in reproductive-age women with systemic lupus erythematosus (SLE) and evaluate its associations with clinical manifestations and disease activity.

**Methods:**

This cross-sectional study enrolled 39 newly diagnosed, treatment-naïve female SLE patients and 37 matched healthy controls. Fasting blood samples were collected in the morning. Serum levels of a comprehensive panel of steroid hormones (including glucocorticoids, mineralocorticoids, androgens, estrogens, and progestogens) were simultaneously quantified using liquid chromatography-tandem mass spectrometry (LC-MS/MS). Clinical parameters and disease activity (SLEDAI-2000) were assessed.

**Results:**

SLE patients exhibited a globally altered steroid hormone profile compared to controls. Levels of key androgens (androstenedione, testosterone, dihydrotestosterone, dehydroepiandrosterone), glucocorticoids (cortisol, corticosterone, cortisone), and mineralocorticoids (aldosterone) were significantly lower in the SLE group. In contrast, serum melatonin was markedly elevated. Analysis across menstrual phases revealed consistently lower progesterone and 17-hydroxyprogesterone, but higher estradiol levels in SLE patients. Correlation analyses demonstrated that lower glucocorticoid and androgen levels were associated with increased inflammatory markers (e.g., lower complement C3/C4, higher IL-6) and hematological abnormalities. Crucially, reduced aldosterone levels correlated with higher 24-hour proteinuria and SLEDAI scores.

**Conclusion:**

Reproductive-age women with SLE display a distinct steroid hormone signature suggestive of hypothalamic-pituitary-adrenal (HPA) axis suppression, characterized by a broad deficiency in adrenal and gonadal steroids alongside hyperestrogenism and elevated melatonin. These hormonal imbalances are significantly correlated with disease activity, inflammation, and renal involvement. The serum steroid hormone profile may serve as a valuable biomarker for assessing SLE activity and provides insights into the endocrine-immune interplay in this disease.

## 1 Introduction

Systemic Lupus Erythematosus (SLE) is a chronic autoimmune disease that affects multiple organs and systems. Its pathogenesis is complex, involving interactions between genetic, environmental, immunological, and endocrine factors. Epidemiological studies reveal a striking gender disparity, with a marked predominance in women of reproductive age and a female-to-male ratio ranging from 9:1 to 10:1 (1) (2). This distinct epidemiological pattern strongly suggests that sex hormones, particularly steroid hormones such as estrogens, progestins, and androgens, play a critical role in the pathogenesis, disease activity, and progression of SLE (3).

Steroid hormones function as crucial endocrine signaling molecules, regulating not only reproductive physiology but also modulating immune system activity. Research indicates that estrogens (e.g., estradiol) can enhance B-cell activation, autoantibody production, and Th2-type immune responses, thereby potentially exacerbating the autoimmune response in SLE (4). Conversely, androgens (e.g., testosterone) and certain progesterone metabolites are thought to exert immunomodulatory or protective effects (5). Women of reproductive age experience dynamic fluctuations in sex hormone levels due to menstrual cycles, pregnancy, and childbirth. These physiological variations represent a critical window during which steroid hormones may influence SLE disease activity and progression.

However, research remains limited and inconsistent regarding the comprehensive serum steroid profile—including estrogens, progestogens, androgens, and their active metabolites—in women of reproductive age with SLE, and its association with specific clinical phenotypes (6). Many studies have limitations, such as a focus on single hormones, inadequate stratification by reproductive age, or small sample sizes, consequently failing to elucidate the overall imbalance of the hormonal network and its precise clinical implications (7). Furthermore, discrepancies between different detection methods (e.g., immunoassays versus mass spectrometry) may also contribute to inconsistent findings (8).

This study aims to precisely characterize the serum steroid hormone profiles in women of reproductive age with SLE compared to healthy controls. By integrating these data with detailed clinical information, we will analyze the correlations between specific hormones—or hormonal patterns—and disease manifestations and severity. Our goal is to identify potential biomarkers, thereby providing a scientific foundation to enhance the understanding of SLE’s sexual dimorphism, improve disease assessment, predict progression, and inform future personalized therapeutic strategies.

## 2 Materials and methods

### 2.1 Study participants

Inclusion criteria: Patients who are newly diagnosed with SLE and have not previously received steroid hormone treatment. Additionally, all patients are women of childbearing age, and patients who are menopausal or perimenopausal are excluded. All patients met the 1997 American College of Rheumatology (ACR) classification criteria for SLE (9). A total of 39 patients were recruited from the Rheumatology and Immunology Department of the First Affiliated Hospital of China Medical University from October 2020 to December 2022 (mean age 28.46 ± 8.92 years).

Exclusion criteria encompassed a history of other autoimmune or endocrine disorders, including rheumatoid arthritis, primary Sjögren’s syndrome, systemic vasculitis, myositis/dermatomyositis, systemic sclerosis, neuropsychiatric disorders, idiopathic thrombocytopenic purpura, primary adrenal diseases, and primary glomerular diseases. Patients with any history of systemic steroid use (within the last 3 months) or hormonal therapy (including oral contraceptives, hormone replacement therapy, or other endocrine treatments) were also excluded from the study. This retrospective study utilized serum samples from 37 premenopausal women (mean age 31.13 ± 7.76 years) as healthy controls (HC). These samples were residual sera obtained from individuals following routine health examinations at the institution’s clinical center. All samples were irreversibly anonymized prior to use. Controls were excluded if they had any autoimmune or endocrine disorders or had undergone an oophorectomy or hysterectomy. This study was conducted in accordance with the Declaration of Helsinki and was approved by the Ethics Committee of the First Affiliated Hospital of China Medical University [Approval No.: 2023-548].

### 2.2 Data collection

#### 2.2.1 Sample collection

Blood samples were collected from all participants (patients and healthy controls) after an overnight fast, between 6:00 AM and 8:00 AM. A 5 mL volume of peripheral venous blood was drawn into plain vacuum tubes. Serum was separated immediately by centrifugation at 3000 rpm for 10 minutes. The supernatant (approximately 2 mL) was aliquoted into sterile microtubes and stored at -80 °C until analysis. Concurrently, general clinical data were collected, including demographic information (sex, age), complete blood count, C-reactive protein (CRP), erythrocyte sedimentation rate (ESR), cytokine and complement levels, and specific autoantibody profiles. Female participants were categorized by menstrual cycle phase (menstrual, follicular, ovulatory, or luteal) at the time of blood draw. Disease activity in SLE patients was assessed using the Systemic Lupus Erythematosus Disease Activity Index 2000 (SLEDAI-2000) score.

#### 2.2.2 Instruments and laboratory analysis

Instrumentation: The following instruments were used in this study: a benchtop low-speed centrifuge (Xiangyi, China); a Legend Micro21R refrigerated microcentrifuge (Thermo Scientific, USA); a Vortex Genie 2 mixer (Scientific Industries, USA); an NDK200-1A 96-well plate nitrogen evaporator (Mio Instrument, China); and a Waters Acquity UPLC-TQS system (Waters, USA).

Serum Sample Preparation: Serum was separated from 5 mL of peripheral blood collected in plain tubes by centrifugation at 3,000 rpm for 10 minutes. For analysis, a 400 μL serum aliquot was mixed with 20 μL of non-derivatized hormone internal standard working solution and vortexed for 5 seconds. Then, 1200 μL of Releasing Agent I was added, and the mixture was vortexed for 5 minutes. After centrifugation at 15,000 rpm and 4 °C for 5 minutes, 1000 μL of the supernatant was transferred and dried under a nitrogen stream at room temperature. The residue was reconstituted in 80 μL of Releasing Agent II, vortexed for 2 minutes, and centrifuged again at 15,000 rpm and 4 °C for 3 minutes. Finally, 60 μL of the resulting supernatant was injected into a 96-well plate for UPLC-TQS analysis.

### 2.3 Statistical analysis

Statistical analyses were performed using SPSS software (version 23.0) and the R programming environment (version 4.2.1). Continuous variables are presented as mean ± standard deviation if normally distributed, and compared between groups using the independent samples t-test. Non-normally distributed continuous variables are expressed as median with interquartile range (IQR) and compared using the Mann-Whitney U test. Correlations were assessed with Pearson’s coefficient for normally distributed data and Spearman’s coefficient for non-normal data. Data visualization was conducted using the ggplot2 package in R. A two-tailed p-value of less than 0.05 was considered statistically significant.

## 3 Results

### 3.1 Demographic data and laboratory findings of patients and controls

The study cohort comprised 39 premenopausal female SLE patients (mean age 28.46 ± 8.92 years) and 37 age-matched, premenopausal healthy controls (mean age 31.13 ± 7.76 years). Participants were stratified according to their self-reported menstrual cycle phase at the time of blood collection. The distribution was as follows: among SLE patients, 6 were in the menstrual phase, 6 in the follicular phase, 8 in the ovulatory phase, and 19 in the luteal phase. Among healthy controls, 6 were in the menstrual phase, 14 in the follicular phase, 6 in the ovulatory phase, and 11 in the luteal phase. Given the known fluctuations in estrogen and progesterone levels across the menstrual cycle, both patient and control groups were stratified into the four aforementioned phases for subsequent analysis to account for this hormonal variability. The detailed group data are presented in **Table 1**.

**Table 1:** Distribution of participants by menstrual cycle phase and age in SLE and HC groups.

Group	Menstrual cycle				Age (years)
	Menstrual phase	Follicular phase	Ovulatory phase	Luteal phase	
SLE Group	6	6	8	19	28.46 ± 8.92
HC Group	6	14	6	11	31.13 ± 7.76

The clinical characteristics of the study participants are summarized in **Supplementary Table 1**. To minimize confounding hormonal variation, perimenopausal and postmenopausal women were excluded from both the case and control groups. Consequently, no significant difference in age was observed between the SLE patient group and the healthy controls (P > 0.05), confirming the comparability of the groups for analysis.

### 3.2 Steroid hormone levels in SLE and HC groups

**Figure 1:**
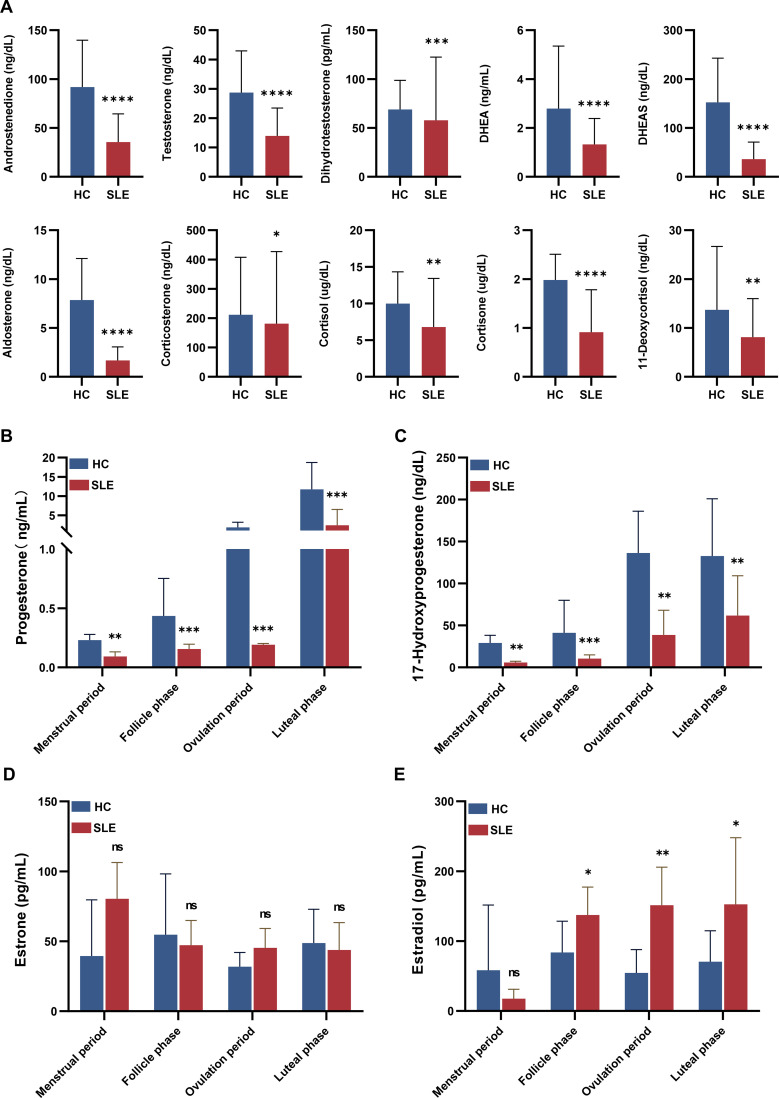
Levels of serum steroid hormones in patients with systemic lupus erythematosus and healthy controls. **(A)** Comparison of Serum Steroid Hormone Levels Between SLE and HC. Comparison of Serum Hormone Levels Between SLE Patients and Healthy Controls Across Menstrual Cycle Phases. **(B)** Progesterone, **(C)** 17-Hydroxyprogesterone, **(D)** Estrone, **(E)** Estradiol. *P < 0.05, **P < 0.01, ***P < 0.001, and ****P < 0.0001.

Statistical analysis was performed using SPSS 23.0. Normality tests indicated that the serum levels of all measured steroids (including melatonin, pregnenolone, testosterone, and others listed) were skewed (P < 0.05 for normality test). Consequently, data are presented as Median ± Interquartile Range (IQR), and between-group differences (SLE vs. controls) were assessed using the non-parametric Mann-Whitney U test. The analysis revealed that serum melatonin levels were significantly elevated in the SLE group compared to controls (P < 0.01). In contrast, the SLE group demonstrated significantly lower serum levels of androstenedione, testosterone, dihydrotestosterone, 11-deoxycortisol, corticosterone, cortisone, cortisol, aldosterone, DHEA-S, and DHEA (all P < 0.05). These results are detailed in **Supplementary Table 2** and illustrated in **Figure 1A**.

### 3.3 Comparison of serum steroid hormone levels with menstrual cycle changes between the SLE and HC group

Consistent with the epidemiology of SLE, both the patient and control groups consisted of premenopausal women. The serum levels of progesterone, 17-hydroxyprogesterone, estrone, and estradiol are known to fluctuate throughout the menstrual cycle. Overall analysis revealed that levels of progesterone and 17-hydroxyprogesterone were significantly lower in the SLE group compared to healthy controls (P < 0.01). In contrast, levels of estrone and estradiol were elevated in the SLE group, though these differences did not reach statistical significance (**Table 2**).

**Table 2:** Comparison of estrogen and progesterone levels between SLE and HC groups.

Hormone	SLE group (n=39)	HC group (n=37)	*P* value
Progesterone (ng/mL)	0.20 (0.02)	0.72 (5.98)	**<0.001**
17-Hydroxyprogesterone (ng/dL)	27.82 (53.78)	57.71 (95.72)	**<0.001**
Estrone (pg/mL)	46.84 ± 24.45	32.99 (42.10)	0.533
Estradiol (pg/mL)	91.98 ± 63.23	82.81 (109.90)	0.568

The bold values indicate that the results are highly statistically significant.

A stratified analysis was performed based on the menstrual cycle phase (menstrual, follicular, ovulatory, and luteal). This analysis revealed that serum progesterone and 17-hydroxyprogesterone levels were significantly lower in the SLE group compared to the control group across all four menstrual phases (P < 0.05). Furthermore, serum estradiol levels were significantly higher in the SLE group during the follicular, ovulatory, and luteal phases (P < 0.05) (**Supplementary Table 3**, **Figures 1B-D**).

### 3.4 Correlation between serum steroid hormones and clinical indicators in SLE

**Figure 2:**
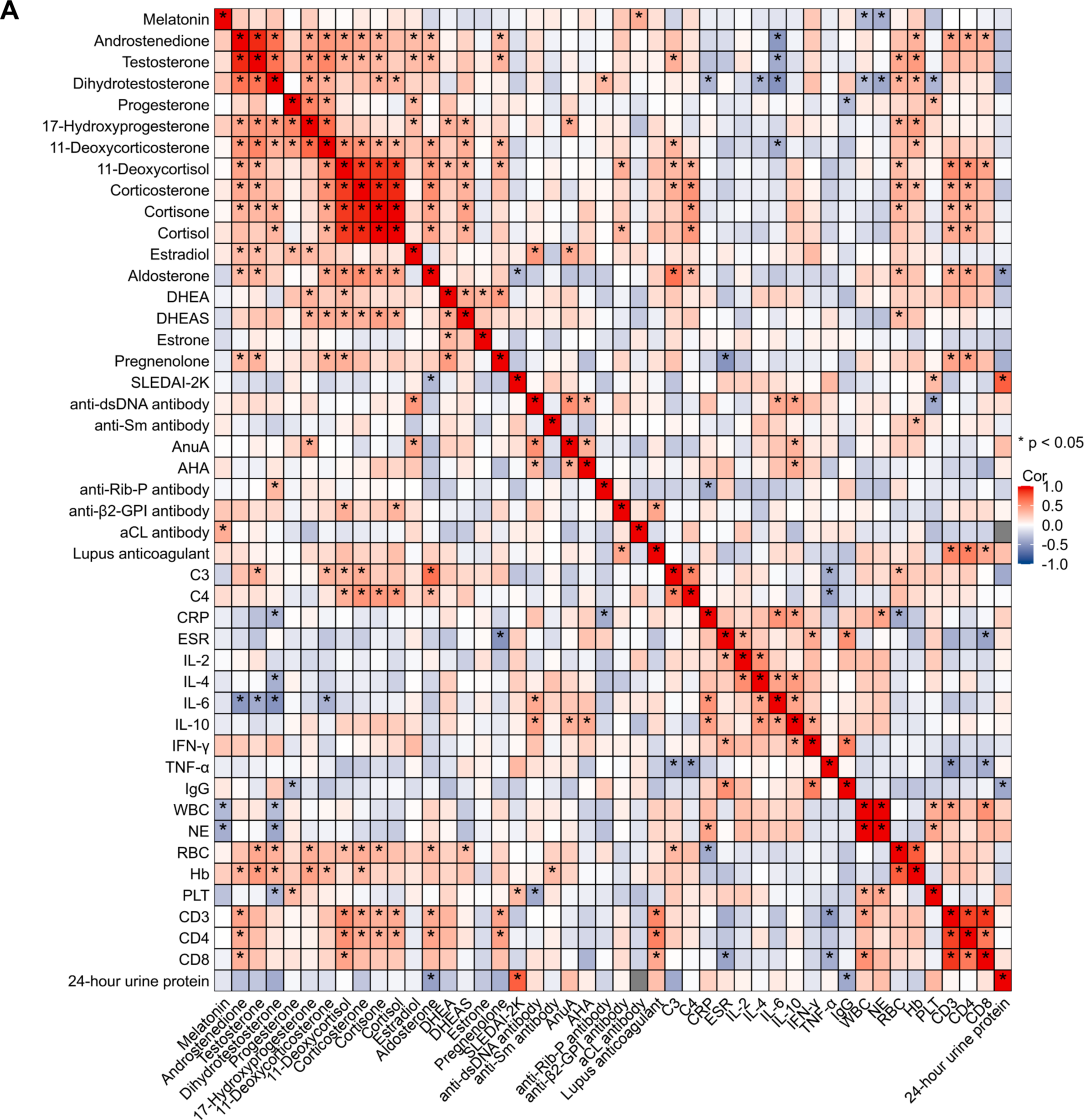
Heatmap of correlations between serum steroid hormones and clinical parameters in female SLE patients.

**Figure 3:**
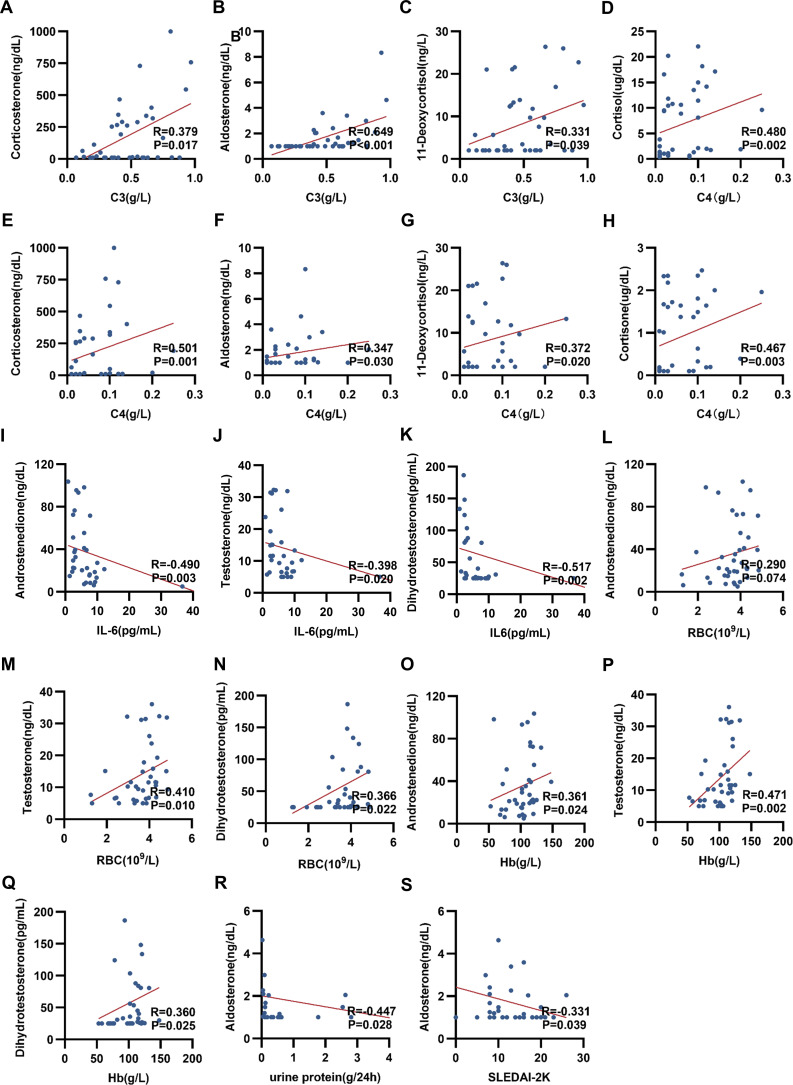
Scatter plots of the correlations between serum steroid hormones and clinical parameters in patients with SLE. Scatter plot of the correlation between C3 levels and **(A)** corticosterone, **(B)** aldosterone and **(C)** 11-deoxycortisol. Scatter plot of the correlation between C4 levels and **(D)** cortisol, **(E)** corticosterone, **(F)** aldosterone, **(G)** 11-deoxycortisol and **(H)** cortisone. Scatter plot of the correlation between the level of Interleukin-6 and **(I)** androstenedione, **(J)** testosterone, and **(K)** dihydrotestosterone. Scatter plot of the correlation between the level of RBC and **(L)** androstenedione, **(M)** testosterone, and **(N)** dihydrotestosterone. Scatter plot of the correlation between hemoglobin levels and **(O)** androstenedione, **(P)** testosterone, and **(Q)** dihydrotestosterone. **(R)** Scatter plot of the correlation between 24-hour proteinuria levels and aldosterone. **(S)** Scatter plot of correlation between SLE disease activity score (SLEDAI-2K) and aldosterone.

Correlation analysis between serum steroid hormone levels and clinical indicators (e.g., hematological parameters, antibody titers, complement C3/C4, CRP, cytokines, immunoglobulins, 24-hour urine protein, and SLEDAI-2K score) was performed using Spearman’s rank correlation. This revealed a complex network of associations (**Figure 2**). Key significant findings include:1. Serum levels of corticosteroids (cortisone, cortisol, 11-deoxycortisol, corticosterone, and aldosterone) showed a positive correlation with complement C3 and C4 levels (P < 0.05; **Figures 3A–H**).2. Androgen levels (androstenedione, testosterone, and dihydrotestosterone) were negatively correlated with interleukin-6 (IL-6) (P < 0.05; **Figures 3I–K**).3. These same androgens demonstrated a positive correlation with erythrocyte and other blood cell counts (P < 0.05; **Figures 3L–Q**).4. Serum aldosterone levels correlated significantly with both 24-hour urine protein and disease activity as measured by the SLEDAI-2000 index (P < 0.05; **Figures 3R, S**).

**Figure 4:**
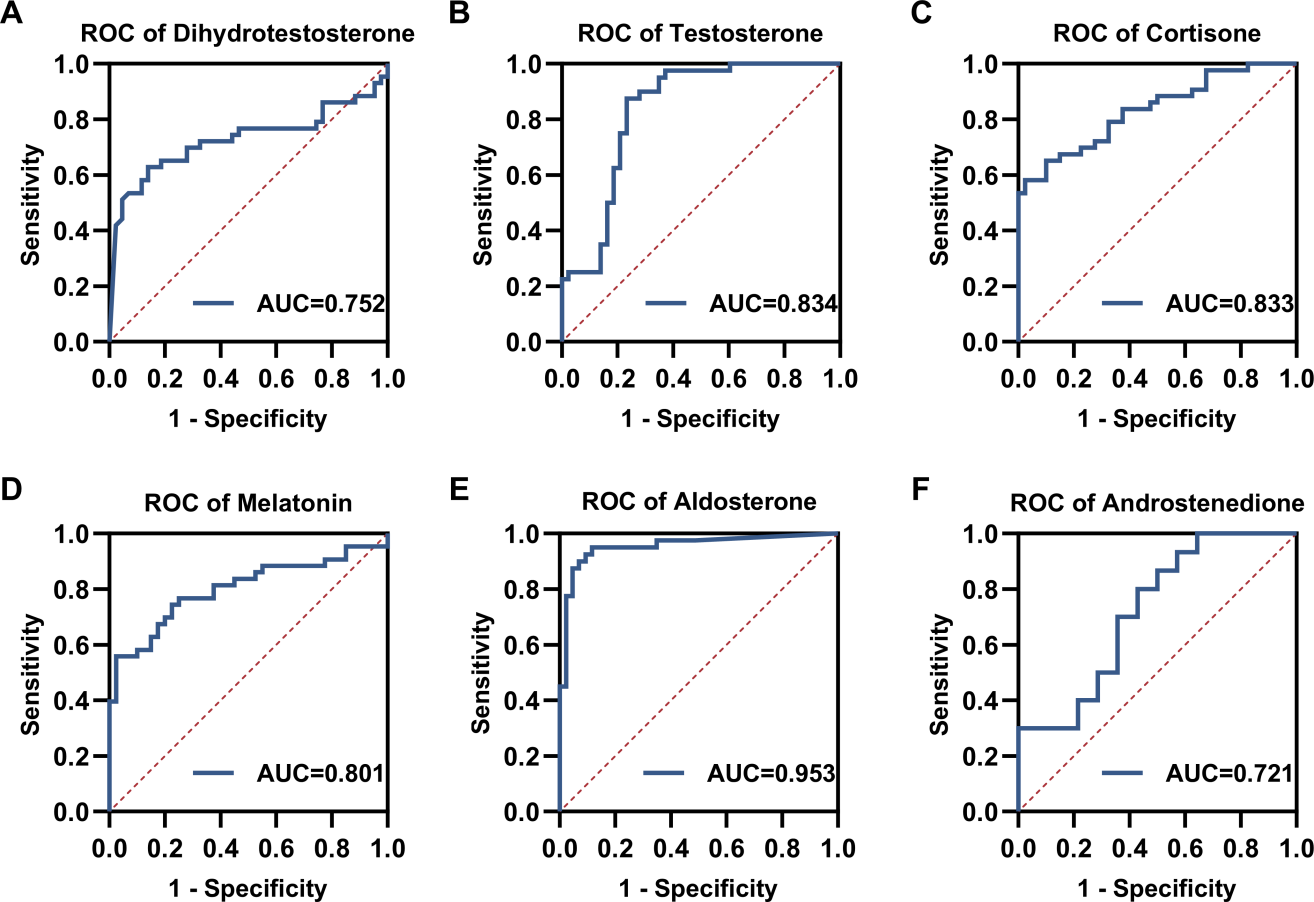
ROC curves of serum steroid hormones for SLE. **(A)** Dihydrotestosterone, **(B)** Testosterone, **(C)** Cortisone, **(D)** Melatonin, **(E)** Aldosterone, **(F)** Androstenedione.

To evaluate the clinical diagnostic potential of these findings, we performed Receiver Operating Characteristic (ROC) curve analysis. The Area Under the Curve (AUC) for serum aldosterone and key androgens suggests these hormones may possess diagnostic utility for SLE (**Figure 4**).

## 4 Discussion

This study delineates a distinct serum steroid hormone profile in reproductive-age women with SLE, characterized by a broad downregulation of adrenal and gonadal steroids, including glucocorticoids, mineralocorticoids, androgens, and progestogens, alongside elevated estradiol and melatonin. These alterations are significantly correlated with key clinical parameters of disease activity and inflammation, suggesting a profound interplay between endocrine function and SLE pathogenesis.

A central finding is the global reduction in adrenal-derived steroids. The decreased levels of cortisol, corticosterone, and aldosterone point toward a state of adrenal insufficiency or HPA axis hypoactivity in SLE. This aligns with the concept that chronic inflammation can dysregulate the HPA axis, leading to an insufficient compensatory hormonal response (10–12). Our data corroborate the work of Straub et al. (13), who attributed similar hormonal reductions in RA and SLE to impaired adrenal synthesis rather than increased clearance. The concurrent elevation of melatonin, an upstream regulator of the HPA axis, may represent a compensatory feedback mechanism attempting to stimulate the blunted axis, or an independent anti-inflammatory response, as suggested by its protective role in lupus-prone mice (14, 15).

Regarding sex hormones, our results confirm the established paradigm of estrogen/progesterone imbalance in SLE (16). The elevated estradiol levels align with its well-documented pro-inflammatory role, which includes promoting B-cell activation and BAFF expression (17–19). Conversely, the significant reduction in progesterone across all menstrual phases is noteworthy. While most evidence, including studies in murine lupus models, positions progesterone as an immunoprotective agent (20–24), its role remains complex. The observed deficiency in our patients could therefore represent a loss of beneficial immunoregulation. However, given the conflicting reports on its potential to enhance IFN-α production (25, 26), it remains unclear whether this hypo-progesteronemia is a cause or a consequence of the disease.

The clinical relevance of this altered steroidome is underscored by our correlation analyses. The positive correlation between glucocorticoid levels (cortisol, corticosterone) and complement C3/C4 suggests that lower adrenal reserve is linked to heightened inflammatory complement consumption. Furthermore, the negative correlation between androgens and IL-6/CRP supports the anti-inflammatory role of androgens, with their deficiency potentially permitting increased pro-inflammatory cytokine activity (27). The positive association of androgens with erythrocyte counts is consistent with their known erythropoietic effects (28, 29), potentially linking hypoandrogenism to SLE-associated anemia.

Notably, we identified a significant inverse correlation between aldosterone and both proteinuria and SLEDAI-2K score. While aldosterone is a known driver of fibrosis in other kidney diseases (30, 31), its low level in active SLE may reflect a protective negative feedback mechanism or simply be part of the global HPA axis suppression. Regardless, this finding supports the clinical utility of RAAS blockade in lupus nephritis (32, 33) and positions serum aldosterone as a potential biomarker for renal activity.

Our study has several limitations. Firstly, the control group in our current study comprised only 39 cases, which limits its classification as a large-scale clinical study. This is because all patients included in our study were newly diagnosed with SLE and had never received any hormone treatment. Such cases are extremely rare in clinical practice. This requirement has enhanced the accuracy and value of our study, while also increasing the difficulty in case collection. At the same time, our testing methods are unprecedented, requiring high technical standards in the laboratory. Therefore, it is difficult to implement them in other medical institutions, and we are temporarily unable to conduct large-scale multi-center studies. Secondly, the cross-sectional nature prevents causal inference regarding whether the hormonal imbalances drive or result from disease activity. Future longitudinal studies in treatment-naïve patients are needed to elucidate the primary nature of these endocrine alterations.

In conclusion, we demonstrate that reproductive-age women with SLE exhibit a pervasive dysregulation of the steroid hormone landscape, likely rooted in HPA axis dysfunction. This endocrine signature is intricately linked to disease activity and specific clinical manifestations. Assessing the serum steroid hormone profile may provide valuable insights into the inflammatory state and disease activity of SLE, opening new avenues for biomarker development and a deeper understanding of the disease’s pathophysiology.

## Data Availability

The original contributions presented in the study are included in the article/**Supplementary Material**. Further inquiries can be directed to the corresponding author.

## References

[1] Tsokos GC . Systemic lupus erythematosus. N Engl J Med. (2011) 365:2110–21. doi: 10.1056/nejmra1100359. PMID: 22129255

[2] Fava A Petri M . Systemic lupus erythematosus: Diagnosis and clinical management. J Autoimmun. (2019) 96:1–13. doi: 10.1016/j.jaut.2018.11.001. PMID: 30448290 PMC6310637

[3] Thanou A Jupe E Purushothaman M Niewold TB Munroe ME . Clinical disease activity and flare in SLE: Current concepts and novel biomarkers. J Autoimmun. (2021) 119:102615. doi: 10.1016/j.jaut.2021.102615. PMID: 33631651 PMC8044029

[4] Petri M . Sex hormones and systemic lupus erythematosus. Lupus. (2008) 17:412–5. doi: 10.1177/0961203308090026. PMID: 18490418

[5] Kokic V Martinovic Kaliterna D Radic M Tandara L Perkovic D . Association between vitamin D, oestradiol and interferon-gamma in female patients with inactive systemic lupus erythematosus: A cross-sectional study. J Int Med Res. (2018) 46(3):1162–1171. doi: 10.1177/0300060517734686. PMID: 29235391 PMC5972245

[6] Nusbaum JS Mirza I Shum J Freilich RW Cohen RE Pillinger MH . Sex differences in systemic lupus erythematosus: Epidemiology, clinical considerations, and disease pathogenesis. Mayo Clinic Proc. (2020) 95(2):384–394. doi: 10.1016/j.mayocp.2019.09.012. PMID: 32029091

[7] Zhong RY . (2019). Effect of Estrogen on Disease Activity and Gut Microbiota in Lupus Mice [master's thesis]. Nanchang University, Nanchang, JS.

[8] Wu G Shen GX Chen YH Xie YC . Discussion on the relationship between serum prolactin and sex hormone levels in patients with systemic lupus erythematosus. J Clin Internal Med. (1999) 16(3):142–143. doi: 10.1016/j.cca.2008.11.009. PMID: 19046958

[9] Hochberg MC . Updating the American College of Rheumatology revised criteria for the classification of systemic lupus erythematosus. Arthritis Rheumatism. (1997) 40:1725. doi: 10.1002/art.1780400928. PMID: 9324032

[10] Sternberg EM Hill JM Chrousos GP Kamilaris T Listwak SJ Gold PW . Inflammatory mediator-induced hypothalamic-pituitary-adrenal axis activation is defective in streptococcal cell wall arthritis-susceptible Lewis rats. PNAS. (1989) 86(7):2374–2378. doi: 10.1073/pnas.86.7.2374. PMID: 2538840 PMC286915

[11] Spies CM Straub RH Cutolo M Buttgereit F . Circadian rhythms in rheumatology--a glucocorticoid perspective. Arthritis Res Ther. (2014) 16(Suppl 2):S3. doi: 10.1186/ar4687. PMID: 25608777 PMC4249493

[12] van der Goes MC Bossema ER Hartkamp A Godaert GL Jacobs JW Kruize AA . Cortisol during the day in patients with systemic lupus erythematosus or primary Sjogren's syndrome. J Rheumatol. (2011) 38(2):285–288. doi: 10.3899/jrheum.100565. PMID: 21159832

[13] Straub RH Weidler C Demmel B Herrmann M Kees F Schmidt M . Renal clearance and daily excretion of cortisol and adrenal androgens in patients with rheumatoid arthritis and systemic lupus erythematosus. Ann Rheumatic Dis. (2004) 63(8):961–968. doi: 10.1136/ard.2003.014274. PMID: 15249323 PMC1755103

[14] Bonomini F Dos Santos M Veronese FV Rezzani R . NLRP3 inflammasome modulation by melatonin supplementation in chronic pristane-induced lupus nephritis. Int J Mol Sci. (2019) 20(14):3466–3479. doi: 10.3390/ijms20143466. PMID: 31311094 PMC6678949

[15] Medrano-Campillo P Sarmiento-Soto H Álvarez-Sánchez N Álvarez-Ríos AI Guerrero JM Rodríguez-Prieto I . Evaluation of the immunomodulatory effect of melatonin on the T-cell response in peripheral blood from systemic lupus erythematosus patients. J Pineal Res. (2015) 58(2):219–226. doi: 10.1111/jpi.12208. PMID: 25612066

[16] Raeisi D Zare ME Nasir A Sherkatolabbasieh H Shafeizadeh S . Sex hormones and prolactin levels and their association with anti cardiolipin antibody in patients with systemic lupus erythematosus. Iranian J Allergy Asthma Immunol. (2018) 17(4):336–345. doi: 10.18502/ijaai.v17i4.93. PMID: 30537797

[17] Drehmer MN Suterio DG Muniz YC de Souza IR Löfgren SE . BAFF expression is modulated by female hormones in human immune cells. Biochem Genet. (2016) 54(5):722–730. doi: 10.1007/s10528-016-9752-y. PMID: 27306360

[18] Rawlings DJ Metzler G Wray-Dutra M Jackson SW . Altered B cell signalling in autoimmunity. Nat Rev Immunol. (2017) 17(7):421–436. doi: 10.1038/nri.2017.24. PMID: 28393923 PMC5523822

[19] Bassi N Luisetto R Ghirardello A Gatto M Valente M Della Barbera M . 17-β-estradiol affects BLyS serum levels and the nephritogenic autoantibody network accelerating glomerulonephritis in NZB/WF1 mice. Lupus. (2015) 24(4-5):382–391. doi: 10.1177/0961203314559636. PMID: 25801881

[20] Cutolo M Straub RH . Sex steroids and autoimmune rheumatic diseases: State of the art. Nat Rev Rheumatol. (2020) 16:628–44. doi: 10.1038/s41584-020-0503-4. PMID: 33009519

[21] Muñoz-Cruz S Togno-Pierce C Morales-Montor J . Non-reproductive effects of sex steroids: Their immunoregulatory role. Curr Top Med Chem. (2011) 11:1714–27. doi: 10.2174/156802611796117630. PMID: 21463251

[22] Tan IJ Peeva E Zandman-Goddard G . Hormonal modulation of the immune system - A spotlight on the role of progestogens. Autoimmun Rev. (2015) 14:536–42. doi: 10.1016/j.autrev.2015.02.004. PMID: 25697984

[23] Hughes GC Martin D Zhang K Hudkins KL Alpers CE Clark EA . Decrease in glomerulonephritis and Th1-associated autoantibody production after progesterone treatment in NZB/NZW mice. Arthritis Rheumatism. (2009) 60(6):1775–1784. doi: 10.1002/art.24548. PMID: 19479860

[24] Papapavlou G Hellberg S Raffetseder J Brynhildsen J Gustafsson M Jenmalm MC . Differential effects of estradiol and progesterone on human T cell activation in vitro. Eur J Immunol. (2021) 51(10):2430–2440. doi: 10.1002/eji.202049144. PMID: 34223649

[25] Elkon KB Stone VV . Type I interferon and systemic lupus erythematosus. J Interferon Cytokine Research: Off J Int Soc For Interferon Cytokine Res. (2011) 31:803–12. doi: 10.1089/jir.2011.0045. PMID: 21859344 PMC3216059

[26] Dosiou C Hamilton AE Pang Y Overgaard MT Tulac S Dong J . Expression of membrane progesterone receptors on human T lymphocytes and Jurkat cells and activation of G-proteins by progesterone. J Endocrinol. (2008) 196(1):67–77. doi: 10.1677/joe-07-0317. PMID: 18180318

[27] Kelly DM Sellers DJ Woodroofe MN Jones TH Channer KS . Effect of testosterone on inflammatory markers in the development of early atherogenesis in the testicular-feminized mouse model. Endocr Res. (2013) 38(3):125–138. doi: 10.3109/07435800.2012.735307. PMID: 23167461

[28] Shahani S Braga-Basaria M Maggio M Basaria S . Androgens and erythropoiesis: Past and present. J Endocrinol Invest. (2009) 32(8):704–716. doi: 10.1007/bf03345745. PMID: 19494706

[29] Latour C Kautz L Besson-Fournier C Island ML Canonne-Hergaux F Loréal O . Testosterone perturbs systemic iron balance through activation of epidermal growth factor receptor signaling in the liver and repression of hepcidin. Hepatology. (2014) 59(2):683–694. doi: 10.1002/hep.26648. PMID: 23907767

[30] Frimodt-Møller M Persson F Rossing P . Mitigating risk of aldosterone in diabetic kidney disease. Curr Opin Nephrol Hypertension. (2020) 29:145–51. doi: 10.1097/mnh.0000000000000557. PMID: 31599747 PMC6903382

[31] Bauersachs J Jaisser F Toto R . Mineralocorticoid receptor activation and mineralocorticoid receptor antagonist treatment in cardiac and renal diseases. Hypertension (Dallas Tex: 1979). (2015) 65:257–63. doi: 10.1161/hypertensionaha.114.04488. PMID: 25368026

[32] Fanouriakis A Kostopoulou M Cheema K Anders HJ Aringer M Bajema I . 2019 update of the Joint European League Against Rheumatism and European Renal Association-European Dialysis and Transplant Association (EULAR/ERA-EDTA) recommendations for the management of lupus nephritis. Ann Rheumatic Dis. (2020) 79(6):713–723. doi: 10.1136/annrheumdis-2020-216924. PMID: 32220834

[33] Tselios K Koumaras C Urowitz MB Gladman DD . Do current arterial hypertension treatment guidelines apply to systemic lupus erythematosus patients? A critical appraisal. Semin Arthritis Rheumatism. (2014) 43(4):521–525. doi: 10.1016/j.semarthrit.2013.07.007. PMID: 23953498

