# Supplementary material for: Serum steroid hormone profiles in reproductive-age women with systemic lupus erythematosus: associations with clinical manifestations and disease activity
Source: Front Immunol. 2026 Apr 22;17:1755060. doi: 10.3389/fimmu.2026.1755060 (PMC13143959; doi:10.3389/fimmu.2026.1755060)
Supplement: Supplementary file 1 [file Table1.docx]

**Supplementary Table S1.** **Clinical Characteristics of the SLE and HC Groups**

| **Characteristic** | **SLE Group (n=39)** | **HC Group (n=37)** |
| --- | --- | --- |
| **Age (years)** | 28.46 ± 8.92 | 31.13 ± 7.76 |
| **WBC (10⁹/L)** | 3.14 (2.38) | - |
| **NE (10⁹/L)** | 2.06 (2.23) | - |
| **RBC (10⁹/L)** | 3.75 (0.91) | - |
| **HGB (g/L)** | 102.33 ± 20.99 | - |
| **PLT (10⁹/L)** | 181.23 ± 65.13 | - |
| **CRP (mg/L)** | 2.9 (4.25) | - |
| **ESR (mm/h)** | 18 (36) | - |
| **IL-2 (pg/mL)** | 2.44 (0) | - |
| **IL-4 (pg/mL)** | 2.44 (0.37) | - |
| **IL-6 (pg/mL)** | 5.93 (5.25) | - |
| **IL-10 (pg/mL)** | 4.8 (10.35) | - |
| **IFN-γ (pg/mL)** | 3.52 (5.86) | - |
| **TNF-α (pg/mL)** | 2.44 (0.99) | - |
| **IgG (g/L)** | 19.07 (8.41) | - |
| **C3 (g/L)** | 0.48 ± 0.23 | - |
| **C4 (g/L)** | 0.04 (0.09) | - |
| **CD4⁺ T cells (10⁹/L)** | 228 (191.5) | - |
| **CD8⁺ T cells (10⁹/L)** | 276.5 (189.75) | - |
| **CD3⁺ T cells (10⁹/L)** | 504 (396.25) | - |
| **24h Urinary Protein (g/24h)** | 0.24 (0.52) | - |
| **dsDNA (IU/mL)** | 74.1 (175) | - |
| **SLEDAI-2K** | 13.72 ± 0.35 | - |
| **Constitutional symptoms (fatigue, fever) [n (%)]** | 15 (38.46) | - |
| **Arthralgia/Arthritis [n (%)]** | 18 (46.15) | - |
| **Myositis [n (%)]** | 2 (5.13) | - |
| **Mucocutaneous involvement [n (%)]** | 22 (56.41) | - |
| **Serositis [n (%)]** | 10 (25.64) | - |
| **Renal involvement [n (%)]** | 13 (33.33) | - |
| **Hematologic involvement [n (%)]** | 21 (53.85) | - |
| **Neuropsychiatric involvement [n (%)]** | 4 (10.26) | - |
| **Cardiac/Vascular involvement [n (%)]** | 8 (20.53) | - |
| **Gastrointestinal involvement [n (%)]** | 6 (15.38) | - |
| **Pulmonary involvement [n (%)]** | 4 (10.26) | - |

**Notes:** Data are presented as **mean ± standard deviation** for normally distributed continuous variables and **median (interquartile range)** for non-normally distributed continuous variables. Categorical data are presented as **n (%)**.

**Abbreviations:** SLE, Systemic Lupus Erythematosus; HC, Healthy Control; WBC, White Blood Cells; NE, Neutrophils; RBC, Red Blood Cells; HGB, Hemoglobin; PLT, Platelets; CRP, C-Reactive Protein; ESR, Erythrocyte Sedimentation Rate; IL, Interleukin; IFN-γ, Interferon-gamma; TNF-α, Tumor Necrosis Factor-alpha; IgG, Immunoglobulin G; C3/C4, Complement 3/4; dsDNA, double-stranded DNA; SLEDAI-2K, Systemic Lupus Erythematosus Disease Activity Index 2000.
