# Supplementary material for: Serum steroid hormone profiles in reproductive-age women with systemic lupus erythematosus: associations with clinical manifestations and disease activity
Source: Front Immunol. 2026 Apr 22;17:1755060. doi: 10.3389/fimmu.2026.1755060 (PMC13143959; doi:10.3389/fimmu.2026.1755060)
Supplement: Supplementary file 2 [file Table2.docx]

**Supplementary Table S2.** **Comparison of Serum Steroid Hormone Profiles between Patients with SLE and HC Groups**

| **Steroid Hormone** | **SLE Group (n=39)** | **HC Group (n=37)** | ***P* value** |
| --- | --- | --- | --- |
| Pregnenolone (ng/dL) | 45.79 (41.36) | 58.17 (59.92) | 0.162 |
| Androstenedione (ng/dL) | 22.33 (31.04) | 76.50 (59.54) | **<0.001** |
| Testosterone (ng/dL) | 10.61 (10.23) | 25.43 (17.12) | **<0.001** |
| Dihydrotestosterone (pg/mL) | 27.64 (43.39) | 68.95 ± 29.69 | **<0.001** |
| 11-Deoxycorticosterone (ng/dL) | 2.07 (2.28) | 1.93 (1.64) | 0.505 |
| Corticosterone (ng/dL) | 20.37 (279.94) | 125.93 (136.47) | **0.023** |
| Aldosterone (ng/dL) | 1.00 (0.86) | 6.94 (4.81) | **<0.001** |
| 11-Deoxycortisol (ng/dL) | 3.40 (10.66) | 9.15 (8.05) | **0.005** |
| 21-Deoxycortisol (ng/dL) | 0.50 (1.57) | 0.52 (0.64) | 0.125 |
| Cortisone (μg/dL) | 0.39 (1.56) | 1.98 ± 0.53 | **<0.001** |
| Cortisol (μg/dL) | 2.48 (10.02) | 9.99 ± 4.31 | **0.007** |
| Dehydroepiandrosterone (ng/mL) | 1.00 (1.46) | 2.07 (1.32) | **<0.001** |
| Dehydroepiandrosterone sulfate (μg/dL) | 27.40 (25.20) | 133.54 (76.18) | **<0.001** |
| Melatonin (pg/mL) | 20.80 (22.61) | 6.78 (8.00) | **<0.001** |

**Notes:**

- Data are presented as **median (interquartile range)** unless otherwise specified. Two values in the HC group (Dihydrotestosterone and Cortisone) are presented as **mean ± standard deviation**, as indicated in the original data.
- **SLE**, Systemic Lupus Erythematosus; **HC**, Healthy Control.
- Bolded *P* values indicate statistical significance (typically *P* < 0.05).
