# Supplementary material for: Serum steroid hormone profiles in reproductive-age women with systemic lupus erythematosus: associations with clinical manifestations and disease activity
Source: Front Immunol. 2026 Apr 22;17:1755060. doi: 10.3389/fimmu.2026.1755060 (PMC13143959; doi:10.3389/fimmu.2026.1755060)
Supplement: Supplementary file 3 [file Table3.docx]

**Supplementary Table S3. Comparison of Estrogen and Progesterone Levels by Menstrual Cycle Phase between SLE and HC Groups**

| **Hormone** | **Menstrual Cycle Phase** | **SLE Group** | **HC Group** | ***P* value** |
| --- | --- | --- | --- | --- |
| **Progesterone (ng/mL)** | Menstrual | 0.11 (0.03) | 0.23 ± 0.05 | **0.002** |
|  | Follicular | 0.16 ± 0.04 | 0.29 (0.32) | **<0.001** |
|  | Ovulatory | 0.19 ± 0.01 | 1.45 (0.11) | **<0.001** |
|  | Luteal | 0.20 (1.55) | 11.74 ± 6.70 | **<0.001** |
| **17-Hydroxyprogesterone (ng/dL)** | Menstrual | 5.13 (0.72) | 29.23 ± 8.92 | **0.002** |
|  | Follicular | 10.55 ± 4.43 | 27.38 (30.75) | **<0.001** |
|  | Ovulatory | 38.59 ± 29.55 | 136.43 ± 49.61 | **0.001** |
|  | Luteal | 61.76 ± 47.46 | 132.82 ± 68.21 | **0.005** |
| **Estrone (pg/mL)** | Menstrual | 80.44 ± 25.88 | 24.30 (11.10) | 0.065 |
|  | Follicular | 47.20 ± 17.76 | 34.75 (41.17) | 0.779 |
|  | Ovulatory | 45.31 ± 13.88 | 31.87 ± 10.14 | 0.059 |
|  | Luteal | 43.86 ± 19.47 | 48.73 ± 24.21 | 0.641 |
| **Estradiol (pg/mL)** | Menstrual | 9.96 (13.97) | 24.41 (9.69) | 0.240 |
|  | Follicular | 137.29 ± 40.12 | 83.82 ± 44.61 | **0.033** |
|  | Ovulatory | 151.44 ± 54.30 | 54.42 ± 33.38 | **0.005** |
|  | Luteal | 152.59 ± 95.47 | 70.58 ± 44.08 | **0.017** |

**Notes:**

- Data are presented as **median (interquartile range)** or **mean ± standard deviation** based on their distribution, as indicated in the original data structure.
- SLE, Systemic Lupus Erythematosus; HC, Healthy Control.
- Bolded *P* values indicate statistical significance (*P* < 0.05).
